# The Small Metal-Binding Protein SmbP Improves the Expression and Purification of the Recombinant Antitumor-Analgesic Peptide from the Chinese Scorpion *Buthus martensii* Karsch in *Escherichia coli*

**DOI:** 10.3390/cimb44020038

**Published:** 2022-01-22

**Authors:** Evelyn Martinez-Mora, Eder Arredondo-Espinoza, Nestor G. Casillas-Vega, Maria Elena Cantu-Cardenas, Isaias Balderas-Renteria, Xristo Zarate

**Affiliations:** 1 Facultad de Ciencias Quimicas, Universidad Autonoma de Nuevo Leon, Av. Universidad s/n, Cd. Universitaria, San Nicolas de los Garza 66455, Mexico; evelyn.martinezmr@uanl.edu.mx (E.M.-M.); eder.arredondosp@uanl.edu.mx (E.A.-E.); maria.cantucd@uanl.edu.mx (M.E.C.-C.); isaias.balderasrn@uanl.edu.mx (I.B.-R.); 2 Departamento de Patologia Clinica, Hospital Universitario Dr. Jose Eleuterio Gonzalez, Universidad Autonoma de Nuevo Leon, Monterrey 64460, Mexico; ncasillasv@uanl.edu.mx

**Keywords:** SmbP, small metal-binding protein, BmK-AGAP, *Escherichia coli*, recombinant peptides, anticancer activity

## Abstract

We have recently shown that SmbP, the small metal-binding protein of *Nitrosomonas europaea*, can be employed as a fusion protein to express and purify recombinant proteins and peptides in *Escherichia coli*. SmbP increases solubility, allows simple, one-step purification through affinity chromatography, and provides superior final yields due to its low molecular weight. In this work, we report for the first time the use of SmbP to produce a recombinant peptide with anticancer activity: the antitumor-analgesic peptide (BmK-AGAP), a neurotoxin isolated from the venom of the Chinese scorpion *Buthus martensii* Karsch. This peptide was expressed in *Escherichia coli* SHuffle for correct, cytoplasmic, disulfide bond formation and tagged with SmbP at the N-terminus to improve its solubility and allow purification using immobilized metal affinity chromatography. SmbP_BmK-AGAP was found in the soluble fraction of the cell lysate. After purification and removal of SmbP by digestion with enterokinase, 1.8 mg of pure and highly active rBmK-AGAP was obtained per liter of cell culture. rBmK-AGAP exhibited antiproliferative activity on the MCF-7 cancer cell line, with a half-maximal inhibitory concentration value of 7.24 μM. Based on these results, we considered SmbP to be a suitable carrier protein for the production of recombinant, biologically active BmK-AGAP. We propose that SmbP should be an attractive fusion protein for the expression and purification of additional recombinant proteins or peptides that display anticancer activities.

## 1. Introduction

The development of new therapeutic agents against cancer has become of primary importance, since cancer is now recognized as the second most-frequent cause of mortality globally, being responsible for around 10 million deaths in the year 2020 [1]. Surgery, chemotherapy, and radiotherapy are the first treatment options, but they present adverse effects due to a lack of selectivity towards cancer cells [2]. Some novel cancer therapies in development are based on toxic peptides isolated from venoms [3]. Scorpion venoms are complex mixtures of aqueous components, including carbohydrates, amino acids, ions, nucleotides, salts, enzymes, and low molecular weight peptides, which may act as toxins, providing specialized weapons for predation and defense [4]. The venom of the Chinese scorpion *Buthus martensii* Karsch (BmK) contains mixtures of peptides that have analgesic and antitumor activities. The antitumor-analgesic peptide (BmK-AGAP) from this organism belongs to a group of long-chain scorpion peptides, and has a molecular weight of 7.1 kDa, comprising 66 amino acids [5,6,7]. Researchers have reported the effect of BmK-AGAP on MCF-7 and MDA-MB-231 cell lines (both derived from human breast adenocarcinoma), observing that the cell viability decreased considerably on treatment [8].

Exploring the therapeutic potential of scorpion toxins, including comprehensive research and their use in clinical trials, is mainly impaired by the low yield of purified toxins from milked venom. Therefore, the production of toxin-derived peptides and proteins, either by heterologous expression or chemical synthesis, is the strategy of choice for research groups and the pharmaceutical industry to overcome this limitation [9]. Recombinant toxins have many applications, including drug development, use as biotechnological tools and bioinsecticides, and anti-venom antibody production. Many toxins from several venom sources have been successfully expressed, most of them in microorganisms, taking advantage of the speed and cost-effectiveness of these expression systems. There is also the possibility of genetically engineering the recombinant product to express the wild-type toxin and variants via site-directed mutagenesis. The most employed microbial systems are those of *Escherichia coli*, *Pichia pastoris*, and *Saccharomyces cerevisiae* [10].

*Escherichia coli*, to this date, is still the first option for heterologous protein and peptide expression and purification. To enhance production and increase solubility and stability of the peptides in this microorganism, fusion proteins have been used extensively. Our research team has previously described a new fusion protein: the small metal-binding protein (SmbP) isolated from the periplasm of the bacterium *Nitrosomonas europaea* [11]. SmbP can bind to metal ions such as Cu(II), Ni(II), and Zn(II), and its biological function is the expulsion of these ions, given that excessive concentrations can be toxic to the cell [12]. The metal-binding capacity of SmbP also allows recombinant proteins to be purified using immobilized metal affinity chromatography (IMAC) [13,14]. An essential advantage provided by SmbP is the enhancement of the final yields due to its low molecular weight (~10 kDa). We have previously reported the expression and purification of bioactive proteins tagged with SmbP, such as the human growth hormone and the antimicrobial peptides (AMPs) Bin1b, VpDef, and LL-37, with satisfactory results [15,16,17,18]. Since the VpDef and Bin1b peptides contain disulfide bonds, the *E. coli* strain SHuffle was used for peptide expression in its cytoplasm; the results showed that SHuffle could properly form these bonds to produce active peptides [16].

Due to the importance of developing robust expression systems to produce biologically active peptides, especially with anticancer activity, here we describe the expression of BmK-AGAP tagged with SmbP to improve its solubility, facilitate its purification, and ultimately produce considerable amounts of a biologically active peptide with antiproliferative properties in vitro against the cancer cell line MCF-7.

## 2. Materials and Methods

### 2.1. Strains, Vectors, and Reagents

The gene for BmK-AGAP was synthesized by GenScript (Piscataway, NJ, USA), and the primers by T4 OLIGO (Irapuato, Guanajuato, Mexico). The vector pET30a(+)_SmbP was previously constructed in our laboratory [11]. *Escherichia coli* SHuffle T7, restriction enzymes, and *Vent* DNA polymerase were purchased from New England Biolabs (Ipswich, MA, USA); T4 DNA ligase from Bio Basic Inc. (Amherst, NY, USA); Enterokinase, His, Bovine from GenScript. MCF-7 cell line (ATCC^®^HTB-22^TM^) was purchased from American Type Culture Collection (Manassas, VA, USA).

### 2.2. Construction of the Expression Vector pET30a(+)_SmbP_BmK-AGAP

Double digestion of the plasmid pUC57_BmK-AGAP was performed using NcoI and XhoI to expose the cohesive ends and release the gene sequence for subsequent ligation into the expression vector pET30a(+)_SmbP. The ligation reaction was carried out with a 1:3 molar ratio (vector:insert) using the enzyme T4 DNA ligase. Rubidium-competent *E. coli* DH5α cells were transformed with 10 µL of the ligation reaction solution. A polymerase chain reaction (PCR) was performed to confirm correct assembly of the plasmid by amplifying the DNA sequence of the *BmK-AGAP* gene using the Green Taq DNA polymerase io Basic Inc. and the primers Forward: 5′ ACGTACGTACCATGGGTGTGCGTGACG 3′; and Reverse: 5′ ACTGACTGACTCGAGTTAGCCACCGTTGCATTTACC 3′. The DNA construct was fully characterized by sequencing.

### 2.3. Expression of SmbP_BmK-AGAP Recombinant Protein

The expression vector pET30a(+)_SmbP_BmK-AGAP was transformed into rubidium-competent *E. coli* SHuffleT7 cells. The clones were grown and selected on LB agar plates with 30 µg/mL kanamycin. The colonies obtained were seeded in LB medium with kanamycin and incubated at 37 °C with shaking (200 rpm) overnight to provide inocula. For small-scale expression, 2 mL of LB medium with kanamycin was inoculated 1:100 with the overnight culture, which was incubated at 37 °C, with shaking at 200 rpm, until it reached an optical density at 600 nm (OD_600_) between 0.4–0.6. Isopropyl-β-thiogalactoside (IPTG) was then added up to a final concentration of 1 mM to induce recombinant protein expression. The incubation was continued for 16 h at 25 °C, with shaking at 200 rpm.

After the culture was centrifuged at 13,000 rpm for 2 min, glass beads (0.1 mm in diameter) and 100 µL of buffer (50 mM Tris-HCl, 500 mM NaCl, pH 8) were added to the cell pellet and it was vortexed for 4 min to lyse the cells. Then it was centrifuged at 12,000 rpm at 4 °C for 10 min and the supernatant was collected for protein analysis by polyacrylamide gel electrophoresis under denaturing conditions (SDS-PAGE). Once recombinant protein expression was confirmed, larger volumes (1 L) of LB culture medium with 30 μg/mL kanamycin were employed for expression in baffled flasks, these were inoculated with an overnight culture and incubated at 37 °C, with shaking at 200 rpm, until an OD_600_ in the range 0.4–0.6 was reached. IPTG was added up to a final concentration of 1 mM to induce expression, and cells were incubated for 16 h at 25 °C.

### 2.4. Purification of SmbP_BmK-AGAP Recombinant Protein

After incubation, the culture medium was centrifuged at 8500 rpm at 4 °C for 10 min to collect the cells. The cell pellet was resuspended in buffer (50 mM Tris-HCl, 500 mM NaCl, pH 8), and the cells were mechanically lysed by stirring at 300 rpm with the glass beads on ice for 1 h. The lysate was centrifuged at 8500 rpm at 4 °C for 20 min, and the pellet was discarded. The recombinant protein was purified with IMAC using the FPLC equipment KTA Prime Plus Ghcare, Chicago, IL, USA), and a HiTrap IMAC FF 1 mL chromatographic column (GE Healthcare, Chicago, IL, USA), functionalized with Ni(II) ions. Elution was performed by gradient with the final buffer (50 mM Tris-HCl, 500 mM NaCl, 200 mM imidazole; pH 8). Elution fractions were collected and analyzed by SDS-PAGE. The Bradford technique was used to quantify the protein concentration.

### 2.5. Enzymatic Removal of Fusion Protein

To excise the peptide from the fusion protein, 25 units of enterokinase were added per mg of recombinant protein. The solution was incubated at 25 °C for 24 h. The peptide was purified by column chromatography, using the ProteIndex HiBond Ni-NTA agarose resin (Marvelgent Biosciences Inc., Scarborough, ON, Canada). The enterokinase reaction solution was added to the column and incubated at 4 °C for 1 h, then the free peptide was eluted by gravity. The eluate was analyzed by Tricine-SDS-PAGE. Purity was determined by densitometry using ImageJ software [19].

### 2.6. WST-1 Assay

MCF-7 cells were seeded in a 96-well plate, each well comprising 5000 cells, using DMEM supplemented with 10% fetal bovine serum. The plate was incubated in a humidified atmosphere (5% CO_2_) at 37 °C for 24 h. Subsequently, rBmK-AGAP was added to the 96-well plate by microdilution, 100 µg/mL being the maximal concentration to be tested, and it was incubated in a humid atmosphere of 5% CO_2_ at 37 °C for 24 h. The culture medium with the peptide then was removed and replaced with 95 µL of fresh culture medium and 5 µL of WST-1 reagent (Roche, Merck, Darmstadt, Germany). Incubation was continued under the same conditions for 2 h. The absorbance at 450 nm was measured using a microplate reader (BioTek ELX800, Winooski, VT, USA). The blank control comprised a culture medium with WST-1, the negative control contained cells, DMEM, and Tris buffer without peptide, and for the positive control, we used 0.1% Triton X-100. The assay was performed in triplicate in three independent experiments.

## 3. Results

**Figure 1:**
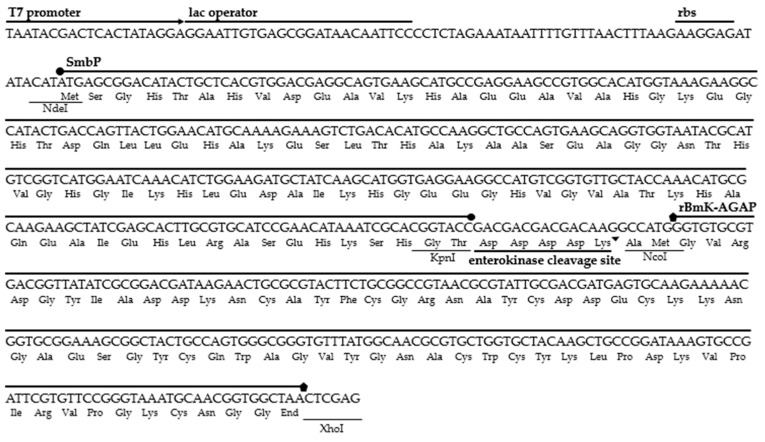
DNA and amino acid sequences for the construct pET30a(+)_SmbP_BmK-AGAP.

The amino acid sequence belonging to the BmK-AGAP peptide was located in the NCBI GenBank database (accession number AAP34332.1). The gene was synthesized and optimized for expression in *E. coli.* We added the restriction sites NcoI at the 5′ end and XhoI at the 3′ end to be inserted downstream of the SmbP fusion protein and the enterokinase cleavage site sequences in the expression vector pET30a(+). The six nucleotides of the NcoI restriction site and two extra nucleotides code for three additional amino acids (Ala, Met, and Gly), these extra nucleotides being added to avoid shifting the open reading frame. Figure 1 details the DNA and amino acid sequences for the SmbP_BmK-AGAP construct.

**Figure 2:**
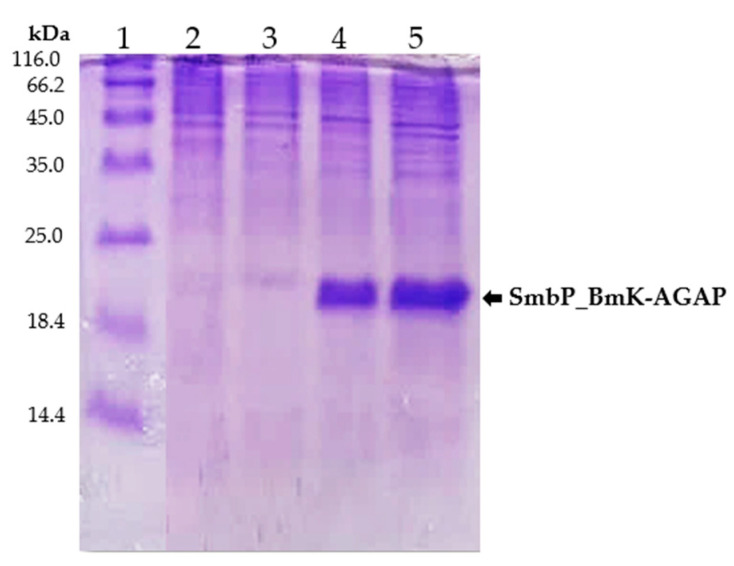
SDS-PAGE (15%) analysis of SmbP_BmK-AGAP small-scale expression. Lane 1: protein marker; Lane 2 and 3: lysates from untransformed and uninduced cells respectively; Lane 4 and 5: lysates from two different colonies of *E. coli* SHuffle cells expressing SmbP_BmK-AGAP (calculated molecular weight: 18.2 kDa).

For small-scale expression experiments, transformed *E. coli* SHuffle cells were incubated overnight at 25 °C after induction with 1 mM IPTG. Cell lysates from induced, untransformed, and uninduced cells were analyzed by protein electrophoresis. Figure 2 shows the 15% SDS-PAGE analysis, a new band at around 18 kDa, corresponding to SmbP_BmK-AGAP, was observed for the induced cells.

**Figure 3:**
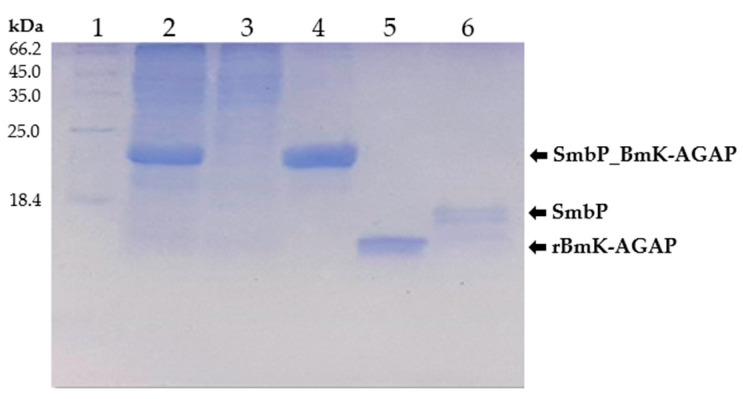
Tricine SDS-PAGE (15%) analysis of SmbP_BmK-AGAP and rBmK-AGAP purification. Lane 1: protein marker; Lane 2: cell lysate; Lane 3: flowthrough; Lane 4: SmbP_BmK-AGAP after IMAC purification; Lane 5: rBmK-AGAP after enterokinase digestion and a second round of IMAC (calculated molecular weight: 7.54 kDa); Lane 6: free SmbP after enterokinase digestion (calculated molecular weight 10.7 kDa).

**Figure 4:**
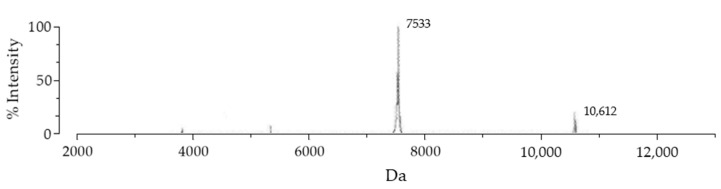
MALDI-TOF MS spectrum of the rBmK-AGAP peptide. After the second purification step, the peptide was analyzed with the VITEK MS (BIOMERIEUX) mass spectrometer using α-Cyano-4-hydroxycinnamic acid as the matrix. The 7533 peak coincided with rBmK-AGAP, while the 10,612 value corresponded to free SmbP present in the sample.

SmbP_BmK-AGAP expression was scaled up to one liter, and purified with IMAC using Ni(II) ions. An imidazole gradient (up to 200 mM) was applied for the elution step. Elution fractions were pooled and protein content was quantified using the Bradford assay. SmbP was removed from BmK-AGAP by digestion with enterokinase and a second round of IMAC, yielding the final product rBmK-AGAP. Tricine SDS-PAGE analysis (Figure 3) includes all the purification steps. As seen in Lane 5, we obtained 98% pure rBmK-AGAP free of SmbP (purity was calculated using the ImageJ software). The peptide was further characterized using MALDI-TOF, Figure 4 shows a *m/z* value of 7533 Daltons which agrees with the calculated molecular mass. We also include the purification summary table for rBmK-AGAP that shows the yields at all purification steps (Table 1).

**Figure 5:**
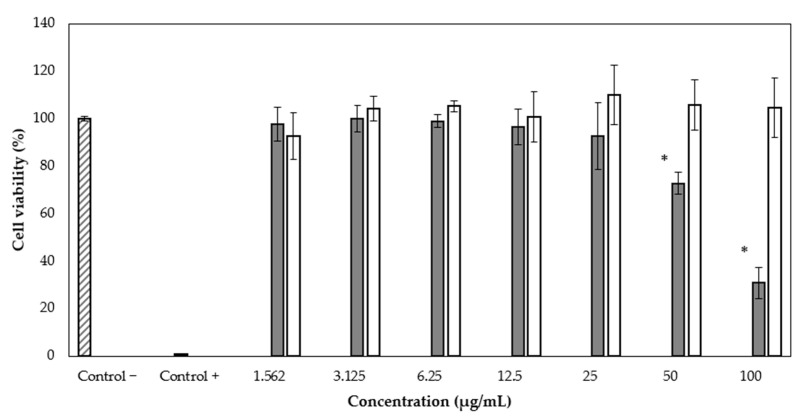
Cytotoxic effect of rBmK-AGAP on the MCF-7 cell line. Viability percentages were determined by the cytotoxicity test using the WST-1 reagent. rBmK-AGAP (gray bar) and SmbP_BmK-AGAP (white bar). * ANOVA considered a value of *p* < 0.05 statistically significant vs. the negative control.

We applied the WST-1 assay to evaluate the antiproliferative activity of rBmK-AGAP on the MCF-7 breast cancer cell line. Different peptide concentrations were used (1.562, 3.125, 6.25, 12.5, 25, 50, and 100 µg/mL), and the complete recombinant protein SmbP_BmK-AGAP was also included, at the same concentrations, to evaluate whether it had any effect on cell viability. After 24 h of treatment, it was observed that rBmK-AGAP inhibits viability in a dose-dependent manner from the second-highest concentration (Figure 5). The half-maximal inhibitory concentration value (IC_50_) of rBmK-AGAP was calculated as 54.6 µg/mL (7.24 µM) using the GraphPad Prism 5.03 program. The complete SmbP_BmK-AGAP construct did not show antiproliferative activity on MCF-7 (Figure 5).

## 4. Discussion

The BmK-AGAP peptide isolated from the Chinese scorpion *Buthus martensii* Karsch is a promising molecule for developing new anticancer therapies [20,21]. Since it is not possible to use the full venom from the scorpion due to its toxicity, and low amounts of the peptide are usually obtained directly from it, heterologous expression systems may be the strategy needed to produce significant amounts of the active peptide. Expression of BmK-AGAP in *E. coli* using different fusion proteins has been previously reported, with mixed results. Here, based on our experience with the carrier protein SmbP and the *E. coli* strain SHuffle to produce soluble, active AMPs, we decided to express and purify BmK-AGAP using a similar strategy.

SmbP has shown to be exceptionally useful for producing recombinant proteins in the periplasm or cytoplasm of *E. coli* that contain disulfide bonds, including biologically active proteins, such as human growth hormone [15] and the AMPs Bin1b and VpDef [16,17]. BmK-AGAP has four disulfide bonds, which are difficult to form in the reducing environment of the bacterial cytoplasm. *E. coli* strains have been genetically modified to change the redox state of the cytoplasm and allow the formation of disulfide bonds and, thus, an adequate folding of protein, including the strain used in this work: *E. coli* SHuffle. This strain has mutations in the thioredoxin reductase (*trxB*) and glutathione reductase (*gor*) genes. This means that the reductive pathways involving these enzymes are no longer functional, facilitating the formation of disulfide bonds. *E. coli* SHuffle also has integrated into its chromosome the disulfide bond isomerase gene (*dsbC*), without its signal sequence, that directs it to the periplasm to perform its activity by adjusting the disulfide bonds that are incorrectly bound, compared to the native protein [22]. We previously reported the complete characterization of the Bin1b peptide, tagged with SmbP and expressed in the cytoplasm in *E. coli* SHuffle, proving the formation of its three disulfide bonds [16]. Therefore, based on this past experience, we expect rBmK-AGAP expressed in *E. coli* SHuffle to contain its disulfide bonds.

SmbP_BmK-AGAP expressed in the cytoplasm was found in the soluble fraction of the cell lysate. This result was expected using SmbP as fusion protein, since we had previously observed soluble expression for the cationic peptides VpDef, Bin1b, and LL-37. The final amount of pure peptide, rBmK-AGAP, after SmbP removal and second IMAC purification, was 1.8 mg per liter of cell culture. Comparing these results with previous reports, we find that the fusion partner, SmbP, shows superior qualities for the production of BmK-AGAP in combination with *E. coli* SHuffle. It has been reported that the solubility of the peptide is considerably lower using a His-tag at the N-terminus and *E. coli* BL21(DE3) for expression; the authors found a greater amount of peptide as inclusion bodies [23]. Recombinant BmK-AGAP, and several mutants, were expressed using the pSYPU vector, which included the *trxA* gene that encodes thioredoxin reductase. This protein catalyzed dithiol-disulfide exchange reactions. When the different peptide mutants were expressed in *E. coli* BL21(DE3), 1–3 mg/L of recombinant peptides were obtained in the soluble fraction [21]. The highest production for BmK-AGAP reported so far is using the small ubiquitin-related modifier (SUMO), combined with a His-tag and expressed in *E. coli* BL21(DE3). The total amount of soluble peptide was larger than that reported here, but there is no mention of an experimental strategy for the formation of the disulfide bonds [24].

As for the rBmK-AGAP biological activity, results from the WST-1 assay indicated that the peptide inhibited cell proliferation of MCF-7 in a dose-dependent manner. At a concentration of 50 µg/mL, a decrease in cell viability was observed up to 72.65 ± 4.66%. At the maximum concentration used (100 µg/mL), the viability was 30.84 ± 6.61%. Since the complete SmbP_BmK-AGAP protein did not show any effects on cell viability, it appeared that the fusion protein interfered with the activity of the peptide. We decided to test the full protein construct since we observed before that SmbP did not completely hinder the activity of the AMPs Bin1b and LL-37 [16,18], and anticipated a similar result. Nevertheless, based on different structures and functions of the cationic peptides and BmK-AGAP, it was not surprising that SmbP inhibited the anti-tumor activity of BmK-AGAP. It is well known that the extract from the venom of *Buthus martensii* Karsch has the ability of inhibiting the growth of MCF-7 cells as well as recombinant BmK-AGAP [20,25]. A recent study showed the cytotoxic effect of recombinant BmK-AGAP on the MCF-7 and MDA-MB-231 cell lines, with IC_50_ of 40 µM and 50 µM vely [8]. In this work, the IC_50_ value for the effect of rBmK-AGAP on MCF-7 cells is 7.24 µM, considerably lower than previously reported.

Methodology for the production of rBmK-AGAP, a promising peptide for new anticancer therapies, based on the use of the protein SmbP as a fusion partner and in combination with *E. coli* SHuffle for protein expression. The obtained recombinant peptide shows a considerably lower IC_50_ on the MCF-7 cell line than previously reported. In summary, the use of SmbP as a carrier protein is a promising biotechnological tool that could facilitate the expression and purification of more biologically active recombinant peptides in *E. coli*.

## Figures and Tables

**Table 1:** Purification summary of rBmK-AGAP.

Purification Step	Total Protein (mg)	Peptide (mg)	Yield (%)	Purity (%)
Lysate	60	7.2	100	12
1° IMAC	6.5	5.6	77.8	87
Cleavage and 2° IMAC	1.8	1.8	25	98

## Data Availability

Not applicable.
